# Considering hounsfield units in native CT- scans for diagnosing spondylodiscitis

**DOI:** 10.1186/s12891-025-09106-9

**Published:** 2025-09-02

**Authors:** Max Prost, Roman Taday, Christian Bernard Matar, David Latz, Christoph Beyersdorf, Melanie Elisabeth Röckner, Joachim Windolf, Max Joseph Scheyerer

**Affiliations:** Department of Orthopaedic and Trauma Surgery, Medical Faculty, University Hospital Düsseldorf, Heinrich-Heine-University Düsseldorf, Moorenstraße 5, Düsseldorf, 40225 Germany

**Keywords:** Spondylodiscitis, Diagnostics, Spinal infections, Houndsfield units

## Abstract

**Background:**

The significance of native Computer tomography (CT) Scans as an alternative diagnostic tool beside Magnetic Resonance Imaging (MRI) for spondylodiscitis is poor according to the current data. CT Scans are currently reserved to analyze the bony destruction and for settings in which performing an MRI is contraindicated. Therefore, the aim of this study was to investigate, whether spondylodiscitis leads to a significant pattern of the density distribution from the affected vertebral bodies and discs measured by Hounsfield Units (HU) in native CT Scans. Such a parameter would be a useful tool to aid in the early diagnosis of spondylodiscitis using CT.

**Methods:**

In a retrospective study we analyzed data from 136 patients, who were treated for spondylodiscitis. Patients who provided MRI- and CT- scans of the spine were included. In axial CT planes HU from the affected intervertebral disc as well as from the affected vertebral bodies and from the unaffected adjacent intervertebral discs and vertebral bodies from the level above and below as reference were measured.

**Results:**

The average measured HU of the affected disc were 26.0% less than in the not affected adjacent discs (*p* < 0.001). The average measured HU of the affected vertebral bodies were 33.77% higher than in the not affected adjacent vertebral body’s (*p* < 0.001)). These findings are independent from the affected part of the spine (e.g. cervical, thoracic or lumbar) and from the degree of bony destruction according to Eysel-Peters classification.

**Conclusion:**

A reduction in the HU of the affected intervertebral disc by approximately 25% and/or an increase in the HU of the affected vertebral bodies by approximately 30% compared to the adjacent intervertebral discs or vertebrae indicates spondylodiscitis even in the early stages without destruction and regardless of the location.

## Introduction

Infections of the spine like spondylitis or spondylodiscitis are rare, but still the third most common form of osteomyelitis. Magnetic Resonance Imaging (MRI) remains the gold standard imaging in depicting spondylodiscitis, offering a high sensitivity and specificity. Nevertheless, acquiring high-resolution images through MRI can sometimes be difficult, due to long acquisition time, patient-related contraindications or other logistic issues like access or availability [1, 2]. Alternatively, native or contrast-enhanced computed tomography (CT) is widely accessible and enables faster assessment than MRI and represents the modality of choice for the detection of differential diagnoses of spondylodiscitis with a higher incidence (e.g. osteoporotic fractures or degenerative osteochondrosis) [3, 4]. Spondylodiscitis is characterized by a heterogenic clinical presentation with non-specific signs and symptoms on presentation especially at the early stage of the disease accompanied by a variety of differential diagnoses and is therefore often recognized and treated late. Recent studies have estimated the delay between the onset of the non-specific symptoms and the diagnosis of spondylodiscitis to an average of 45 days [5]. This leads to an increased morbidity of the disease and can even become life threatening [6]. During the early stage of disease in some cases conventional imaging modalities like x-ray can fail to detect subtle changes in vertebral and non-bony surrounding structures [7]. Actually, MRI is known as the best diagnostic tool especially for early spondylodiscitis [6]. But even in MRI it could be difficult to distinguish in early state of the disease between osteochondrosis and spondylodiscitis or between spinal neoplasms and spondylodiscitis [8–12]. Additionally, the indication for obtaining early MRI in a patient with back pain is tied to reasonable suspicion of spondylodiscitis or red flags [8, 13]. In terms of initial diagnostic imaging for spondylodiscitis, CT scans are currently reserved to analyze the bony destruction and for settings in which performing an MRI is contraindicated, and more expensive nuclear imaging (PET/CT) is not feasible [8, 14, 15]. This is caused by the fact, that its accuracy remains significantly lower than MRI and PET/CT, particularly in early stages. CT is currently used mostly for percutaneous needle biopsy and drainage of abscesses [6, 8, 16, 17]. Nevertheless, measuring local bone quality using CT scans with Hounsfield units (HU) quantification is possible and has been shown to be a reliable method to assess bone density changes for osteoporosis, spondylarthritis and osteochondrosis across all vertebrae [18–21].

HU is a quantitative scale for describing radiodensity which is frequently used in CT scans. In this scale the radiodensity distilled water at standard pressure and temperature is defined as 0 HU, while the radiodensity of air at standard pressure and temperature is defined as − 1000 HU [22, 23]. Exact HU dynamics can vary from one CT acquisition to another due to CT acquisition and reconstruction [24]. 

The aim of the present study was to analyze an alternative method to diagnose spondylodiscitis with a native CT scan. To assess this aim, we want to show whether the measured HU in the infected intervertebral discs and vertebral bodies, in comparison to adjacent non infected spine segments display a significant pattern to be interpreted as a reliable parameter that can predict an infectious entity of the spine. Such a parameter would be a useful tool to aid in the early diagnosis of spondylodiscitis using CT.

## Patients and Methods

We performed a retrospective single center data analysis. An existing database of patients who were treated with spondylodiscitis in our institution from 2014 to 2022 was screened for patients who met our inclusion criteria. We included patients in whom the diagnosis of spondylodiscitis was ensured by MRI, laboratory examination and positive pathogen detection whether with needle guided or intraoperative biopsy or with blood culture and who had a CT scan of the affected spine segment. Patients with tuberculous spondylodiscitis were excluded from our investigation. Patients who did not have an MRI of the spine and patients without a CT or with an incomplete CT examination of the spine were excluded. The maximal accepted time between MRI an CT scan was 14 days, if the time between imaging was longer the patients were excluded. Further, we excluded patients from the study who developed sondylodsicitis after spinal surgery in which implants such as screws, rods or cages were inserted, provided that these implants were still in the body.

**Fig. 1:**
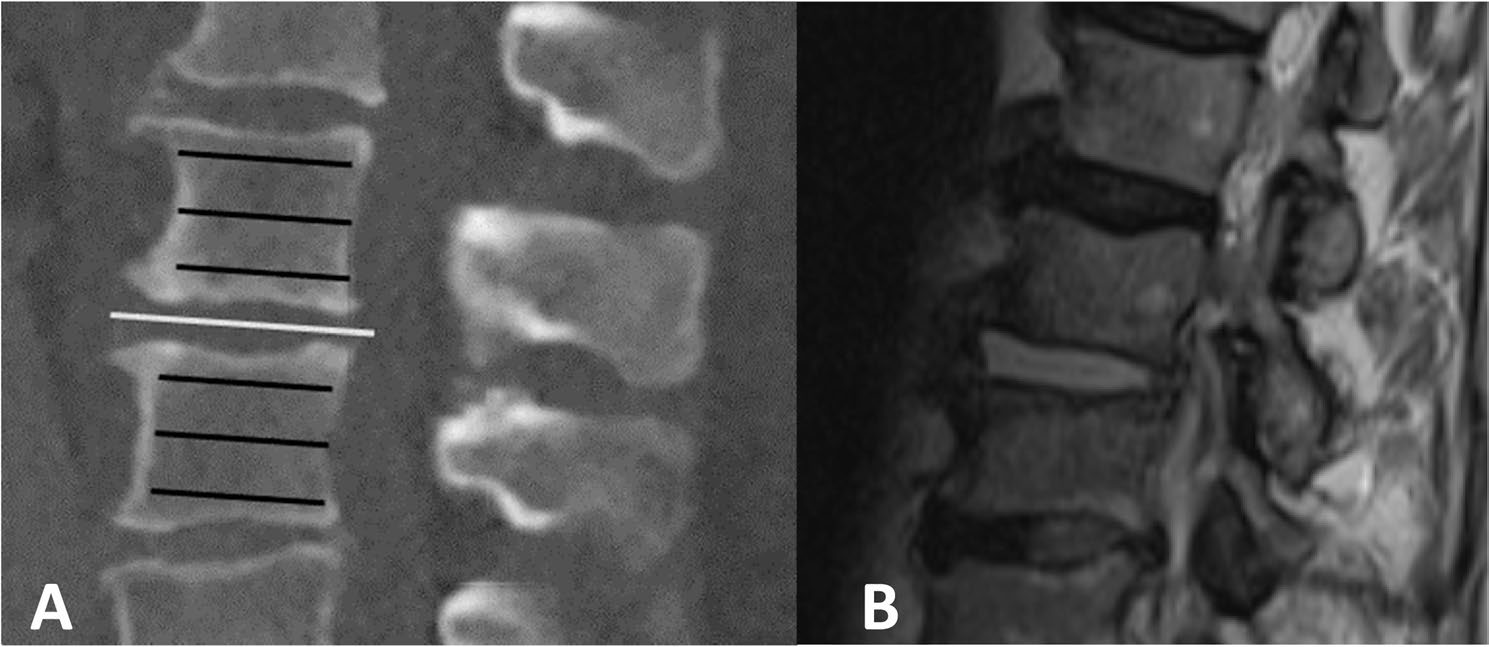
Localization of the performed measurements. **A** shows a lateral reconstruction of a lumbar spine CT Scan. In the vailable T2 MRI Sequence (**B**) of this patient the intervertebral disc between L2 and L3 showed inflammatory changes. This disc was classified in the CT (**A**) as an affected disc (white bar). The intervertebral disc one segment above and one segment below served as reference. The black bars show the localization of the performed measurements in the vertebral bodies which were affected by the spondylodiscitis. The unaffected adjacent vertebral bodies from the level above and below as reference

**Fig. 2:**
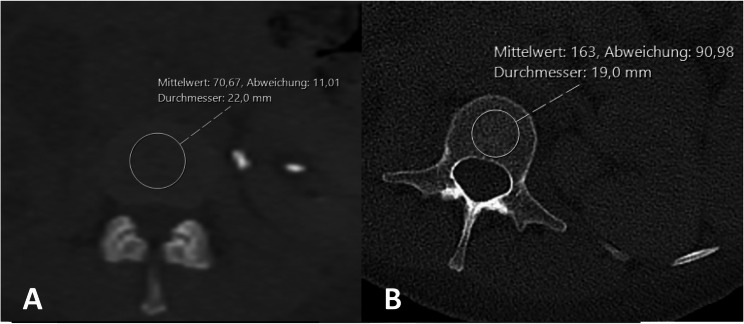
Measurement of the HU in the vertebral discs and the vertebral bodies. Axial reconstruction of a lumbar spine CT Scan. A circular region of interest was selected based on manually defined reference lines, having a diameter of app. 75% of the anteroposterior and transverse diameters of the vertebral body or the intervertebral disc. The average HU within the sample region was displayed by the software (Mittelwert). A shows exemplary a performed measurement in a vertebral disc – HU 70.67. B shows a performed measurement in a vertebral body – HU 163

Demographical data like sex and age as well as data according to the localization of the spondylodiscitis were recorded. The radiological degree of destruction caused by the spondylodiscitis was detected and classified according to the Eysel- Peters Classification [25]. The native spinal CT and MRI were analyzed by the IDS 7-PACS^®^-System (Sectra, Linköping, Sweden). The affected intervertebral discs and vertebral bodies were identified in the available MRI of the patients. For HU measurement we used a three- dimensional multiplanar reconstruction of the respective spine- CT. To analyze the changes of the intervertebral disc, we measured the HU in axial CT- planes from the affected intervertebral disc (identified in the MRI) and from the adjacent unaffected intervertebral discs one level above and below who served as referende (Figs. 1 and 2). For analysis of the vertebral body, we measured the HU from the vertebral bodies which were affected by the spondylodiscitis (above and below the affected intervertebral disc) and the HU from the unaffected adjacent vertebral bodies from the level above and below as reference. HU measurement of the vertebral bodies was taken at from three cross-sectional slices at the level of the cover plate, in the middle of the vertebral body and close to the ground plate (Fig. 1).

The mean of these three measurements was calculated and analyzed. In the axial CT- planes a circular region of interest was selected based on manually defined reference lines, having a diameter of app. 75% of the anteroposterior and transverse diameters of the vertebral body or the intervertebral disc (Fig. 2).

The average HU within the sample region was displayed by the software. The measurements were performed according to the technique described by Schreiber et al. in 2011 [14]. 

### Statistical analysis

Statistical analysis was performed by SPSS^®^ 27 (IBM, Armonk, USA). Descriptive data are given as mean and standard deviation (SD). We tested all continuous variables for normal distribution by Kolmogorov– Smirnov test. Variables that showed normal distribution were analyzed by t-test and variables, which showed no normal distribution, were analyzed by Wilcoxon signed-rank test.

This study was approved by the local ethics committee (Register number 2020 − 914_1) and was conducted according to the revised Declaration of Helsinki.

## Results

From the 246 patients with spondylodiscitis in our database we identified 136 patients who met our inclusion criteria. 110 patients could not be included due to incomplete set of radiological data or due to postoperative spondylodiscitis.

44 patients were female (32.4%), 92 patients were male (67.6%). The average age of the patients was 66.86 (13.08) years. 9 (6.6%) patients had a spondylodiscitis located in the cervical spine, 50 (36.8%) were located in the thoracic and 77 (56.6%) in the lumbar spine.

According to the Eysel-Peters Classification 57 (41.9%) patients were classified as type 1, 55 (40.4%) as type 2 and 24 (17.6%) as type 3.

The average measured HU of the affected disc were 26.0% less than in the unaffected adjacent discs (73.4(29.2) vs. 99.1 (26.5)). The difference was significant (*p* < 0.001).

The average measured HU of the affected vertebral bodies were 33.8% higher than in the unaffected adjacent vertebral body’s (246.8 (81.2) vs. 184.5 (64.7). The difference was significant (*p* < 0.001).

In a subgroup analysis in which we analyzed the difference of the measured HU from the affected discs and vertebral bodies depending on the Eysel-Peters Classification, the measured HU of the affected disc were significant less than in the unaffected adjacent discs independent of the Eysel-Peters type. The measured parameters for the discs were shown in Table 1.

**Table 1:** Measured HU of the affected and unaffected adjacent vertebral disc depending on the Eysel- Peters classification

	Affected disc Type EP 1	Unaffected adjacent disc Type EP 1	Affected disc Type EP 2	Unaffected adjacent disc Type EP 2	Affected Disc Type EP 3	Unaffected adjacent disc Type EP 3
Mean	71.9	96.6	74.7	102.5	73.9	97.4
SD	30.3	23.2	29.9	30.4	26.0	24.2
Significance	*p* < 0.001		*p* < 0.001		*p* < 0.05	

The measured HU of the affected vertebral bodies were significantly higher than in the unaffected adjacent vertebral bodies in patients with a spondylodiscitis type Eysel-Peters 1 and 2. In the patients with a type 3 spondylodiscitis, the difference was not significant. The measured parameters for the vertebral bodies were shown in Table 2.

**Table 2:** Measured HU of the affected and unaffected adjacent vertebral bodies (VB) depending on the Eysel- Peters classification

	Affected VB Type EP 1	Unaffected adjacent VB Type EP 1	Affected VB Type EP 2	Unaffected adjacent VB Type EP 2	Affected VB Type EP 3	Unaffected adjacent VB Type EP 3
Mean	236.0	178.1	241.4	184.6	284.7	199.2
SD	93.1	68.6	67.3	63.5	71.5	57.7
Significance	*p* < 0.001		*p* < 0.001		*p* > 0.05	

In a further subgroup analysis, we analyzed the difference of the measured HU from the affected discs and vertebral bodies depending on the localization of the spondylodiscitis. The measured HU of the affected discs were significantly less than in the unaffected adjacent discs independent of the localization. The measured parameters for the discs were shown in Table 3.

**Table 3:** Measured HU of the unaffected and not affected adjacent vertebral disc depending on localization

	Affected disc cervical spine	Unaffected adjacent disc cervical spine	Affected disc thoracic spine	Unaffected adjacent disc thoracic spine	Affected disc lumbar spine	Unaffected adjacent disc Lumbar spine
Mean	94.7	115.4	81.2	108.5	63.0	87.7
SD	33.5	21.3	31.5	26.9	24.7	19.4
Significance	*p* < 0.005		*p* < 0.001		*p* < 0.001	

The measured HU of the affected vertebral bodies were significantly higher than in the unaffected adjacent vertebral bodies independent of the localization. The measured parameters for the vertebral bodies were shown in Table 4.

**Table 4:** Measured HU of the affected and unaffected adjacent vertebral bodies (VB) depending on the localization

	Affected VB cervical spine	Unaffected adjacent VB cervical spine	Affected VB thoracic spine	Unaffected adjacent VB thoracic spine	Affected VB lumbar spine	Unaffected adjacent VB Lumbar spine
Mean	362.4	271.3	266.9	81.6	211.4	166.6
SD	73.2	50.5	195.6	62.4	64.6	60.5
Significance	*p* < 0.05		*p* < 0.05		*p* < 0.001	

## Discussion

Through the analysis of CT scans from 136 patients, this study demonstrates that the average measured HU of the affected intervertebral disc was 26.0% lower than that of the unaffected adjacent discs (*p* < 0.001). The average measured HU of the affected vertebral bodies was 33.77% higher than that of the unaffected adjacent vertebral bodies (*p* < 0.001). These findings were independent of the affected spinal segment (e.g., cervical, thoracic, or lumbar spine) and the degree of bone destruction according to the Eysel-Peters classification.

MRI remains the gold standard in the early detection of spondylodiscitis due to its high sensitivity and specificity [14, 26]. However, CT imaging is more widely available and remains the first-line imaging modality for patients presenting with unspecific spinal symptoms in depicting differential diagnoses, particularly when MRI is contraindicated or unavailable [8]. A justified indication for obtaining early MRI in a patient with back pain is obligatory tied to a reasonable suspicion of spondylodiscitis or red flags, due to high cost, long acquisition time or patient- related contraindications (non-MR-capable pacemakers, other patient-dependent factors) [8, 13, 26]. Although inaccuracies related to geometry and basic radiation physics principles (i.e. noisy images, variability in axial slices) exist, the value of the HU measurements appear to be a reliable predicting bone mineral density changes of trabecular bone and the density of intervertebral disc [19, 20, 27]. Prior studies have demonstrated that HU values in CT imaging positively correlate with bone mineral density, especially of vertebral endplates [19, 20, 27, 28]. Our findings align with these studies and add further evidence that HU changes in both vertebral bodies and intervertebral discs can serve as indicators of pathological changes in spondylodiscitis. The presence of spondylodiscitis is often indistinguishable from osteochondrosis on native radiologic imaging [15, 29]. Previous studies have shown inconsistent changes in HU values in degenerative disc disease, with a tendency toward reduced HU values in adjacent vertebral bodies as degeneration progresses, supporting the value of HU differentiation in inflammatory versus degenerative conditions [21, 27, 29–32].

Compared to the unaffected adjacent vertebral sections, the observed decrease of HU values of the affected discs accompanied by increased HU values of the affected vertebral bodies, indicate a pathophysiological pattern of disc destruction with secondary reactive osteosclerosis of the adjacent vertebral bodies in spondylodiscitis. These findings were particularly evident in early disease stages like Eysel I and II, likely corresponding to infection-triggered inflammatory processes and reactive bone marrow changes [33]. Due to the fact, that especially in this early stage of the disease, it could be difficult to distinguish between osteochondrosis and spondylodiscitis even in an MRI, the results of our investigation present an important diagnostic tool to improve diagnosing spondylodiscitis. In contrast, in more advanced stages (e.g., Eysel III), the variability and extent of vertebral destruction and reactive bracing bone formations may lead to a heterogeneous HU distribution, which may explain the reduced statistical significance of HU differences in later disease stages. A major strength of this study is the relatively large cohort and standardized analysis of HU values across different spinal segments and disease severities. The study presents a novel, quantitative, and widely available diagnostic indicator that may assist in the early detection of spondylodiscitis.

It must be emphasized, however, that this study only included patients with pyogenic spondylodiscitis. Therefore, the results observed cannot necessarily be extrapolated to other forms of spondylodiscitis, particularly those caused by tuberculosis. This is particularly due to the fact that spondylodiscitis caused by tuberculosis usually results in bone destruction without new bone formation, whereas pyogenic spondylodiscitis is usually accompanied by new bone formation after bone destruction.

However, this is a retrospective, single-center study, which limits the generalizability of the results. There was no direct comparison group with osteochondrosis, and no conclusions can be drawn regarding diagnostic accuracy measures such as sensitivity or specificity.

However, these findings support the hypothesis that HU measurements on native CT scans can help differentiate early spondylodiscitis from degenerative changes, particularly when MRI is not feasible. In such cases, the combination of reduced disc HU values, increased vertebral body HU values, and elevated inflammatory markers as well as clinical red flags (e.g., CRP, leukocytosis, ESR) [6, 8, 15] may strengthen the clinical suspicion of spondylodiscitis and justify expedited MRI diagnostics, thereby potentially reducing delays in diagnosis and treatment initiation.

## Conclusion

In conclusion, native CT imaging may serve as a valuable adjunct in the early diagnosis of spondylodiscitis, especially in settings where MRI is unavailable or contraindicated. A reduction of HU values in the intervertebral disc by approximately 25% and/or an increase of HU values in the vertebral body by approximately 30% compared to adjacent levels may suggest spondylodiscitis. These findings are independent of the spinal region or extent of bone destruction. We recommend prospective studies to validate these HU thresholds, evaluate diagnostic accuracy, and directly compare HU changes in spondylodiscitis versus degenerative diseases. Integrating CT-based HU analysis with clinical and laboratory data may expedite diagnosis and optimize patient care.

## Data Availability

No datasets were generated or analysed during the current study.

## Abbreviations

MRI: Magnetic Resonance Imaging
CT: Computer tomography
PET/CT: Positron Emission Tomography
SEM: Standard error of mean
HU: Hounsfield Units
SD: Standard deviation
STIR: Short- Tau- Inversion- Recovery
VB: Vertebral Body
